# Supplementary material for: Modeling glioblastoma heterogeneity as a dynamic network of cell states
Source: Mol Syst Biol. 2021 Sep 16;17(9):e10105. doi: 10.15252/msb.202010105 (PMC8444284; doi:10.15252/msb.202010105)
Supplement: Supplementary file 6 — Source Data for Figure 5 [file MSB-17-e10105-s004.zip › Figure5A_sourcedata/GSEA_3071/hallmarks_stateIV.GseaPreranked.1623742551702/HALLMARK_APICAL_JUNCTION.html]

Details for gene set HALLMARK\_APICAL\_JUNCTION[GSEA]

|  || Dataset | state53071 |
| Phenotype | NoPhenotypeAvailable |
| Upregulated in class | na\_neg |
| GeneSet | HALLMARK\_APICAL\_JUNCTION |
| Enrichment Score (ES) | -0.38946646 |
| Normalized Enrichment Score (NES) | -1.9964063 |
| Nominal p-value | 0.0 |
| FDR q-value | 0.001760449 |
| FWER p-Value | 0.017 |
Table: GSEA Results Summary

  

Fig 1: Enrichment plot: HALLMARK\_APICAL\_JUNCTION      
 Profile of the Running ES Score & Positions of GeneSet Members on the Rank Ordered List

  

| PROBE | GENE SYMBOL | GENE\_TITLE | RANK IN GENE LIST | RANK METRIC SCORE | RUNNING ES | CORE ENRICHMENT || 1 | LIMA1 |  |  | 58 | 1.805 | 0.0153 | No |
| 2 | NRXN2 |  |  | 83 | 1.621 | 0.0344 | No |
| 3 | GNAI2 |  |  | 340 | 0.980 | -0.0005 | No |
| 4 | LAMB3 |  |  | 413 | 0.892 | -0.0013 | No |
| 5 | MDK |  |  | 495 | 0.819 | -0.0048 | No |
| 6 | ARHGEF6 |  |  | 503 | 0.815 | 0.0058 | No |
| 7 | CADM3 |  |  | 507 | 0.812 | 0.0171 | No |
| 8 | DLG1 |  |  | 520 | 0.798 | 0.0264 | No |
| 9 | CDH4 |  |  | 610 | 0.721 | 0.0199 | No |
| 10 | ADAM23 |  |  | 710 | 0.654 | 0.0104 | No |
| 11 | JAM3 |  |  | 712 | 0.652 | 0.0198 | No |
| 12 | GAMT |  |  | 747 | 0.634 | 0.0225 | No |
| 13 | SHROOM2 |  |  | 828 | 0.589 | 0.0158 | No |
| 14 | PIK3R3 |  |  | 852 | 0.578 | 0.0198 | No |
| 15 | PKD1 |  |  | 894 | 0.563 | 0.0202 | No |
| 16 | CTNNA1 |  |  | 909 | 0.557 | 0.0256 | No |
| 17 | ADAM9 |  |  | 984 | 0.528 | 0.0192 | No |
| 18 | KCNH2 |  |  | 1000 | 0.522 | 0.0239 | No |
| 19 | FSCN1 |  |  | 1003 | 0.521 | 0.0312 | No |
| 20 | EVL |  |  | 1047 | 0.506 | 0.0303 | No |
| 21 | INSIG1 |  |  | 1317 | 0.436 | -0.0150 | No |
| 22 | SIRPA |  |  | 1408 | 0.415 | -0.0262 | No |
| 23 | AKT3 |  |  | 1417 | 0.412 | -0.0218 | No |
| 24 | NLGN2 |  |  | 1534 | 0.381 | -0.0385 | No |
| 25 | NFASC |  |  | 1618 | 0.364 | -0.0491 | No |
| 26 | PTK2 |  |  | 1631 | 0.362 | -0.0461 | No |
| 27 | PTEN |  |  | 1652 | 0.358 | -0.0447 | No |
| 28 | AKT2 |  |  | 1663 | 0.356 | -0.0414 | No |
| 29 | SYMPK |  |  | 1763 | 0.337 | -0.0555 | No |
| 30 | NLGN3 |  |  | 1785 | 0.333 | -0.0547 | No |
| 31 | EXOC4 |  |  | 1852 | 0.323 | -0.0626 | No |
| 32 | CDH6 |  |  | 1920 | 0.310 | -0.0709 | No |
| 33 | PARD6G |  |  | 2109 | 0.282 | -0.1029 | No |
| 34 | RASA1 |  |  | 2437 | -0.262 | -0.1620 | No |
| 35 | HRAS |  |  | 2645 | -0.289 | -0.1975 | No |
| 36 | MPZL1 |  |  | 2678 | -0.292 | -0.1994 | No |
| 37 | ITGB1 |  |  | 2817 | -0.308 | -0.2214 | No |
| 38 | NECTIN2 |  |  | 3006 | -0.333 | -0.2527 | No |
| 39 | TJP1 |  |  | 3113 | -0.348 | -0.2679 | No |
| 40 | BAIAP2 |  |  | 3130 | -0.351 | -0.2659 | No |
| 41 | PARVA |  |  | 3438 | -0.396 | -0.3191 | No |
| 42 | MSN |  |  | 3454 | -0.400 | -0.3161 | No |
| 43 | ACTB |  |  | 3512 | -0.412 | -0.3210 | No |
| 44 | SDC3 |  |  | 3547 | -0.419 | -0.3214 | No |
| 45 | IRS1 |  |  | 3584 | -0.424 | -0.3222 | No |
| 46 | IKBKG |  |  | 3658 | -0.437 | -0.3298 | No |
| 47 | CD99 |  |  | 3752 | -0.456 | -0.3410 | No |
| 48 | TUBG1 |  |  | 3775 | -0.460 | -0.3385 | No |
| 49 | AMIGO2 |  |  | 3965 | -0.502 | -0.3675 | No |
| 50 | HADH |  |  | 4009 | -0.514 | -0.3682 | No |
| 51 | YWHAH |  |  | 4032 | -0.521 | -0.3648 | No |
| 52 | ADAMTS5 |  |  | 4047 | -0.525 | -0.3598 | No |
| 53 | ZYX |  |  | 4087 | -0.536 | -0.3595 | No |
| 54 | MYL12B |  |  | 4093 | -0.539 | -0.3525 | No |
| 55 | RSU1 |  |  | 4127 | -0.546 | -0.3509 | No |
| 56 | ARPC2 |  |  | 4173 | -0.560 | -0.3513 | No |
| 57 | VASP |  |  | 4245 | -0.578 | -0.3565 | No |
| 58 | MAP3K20 |  |  | 4308 | -0.599 | -0.3597 | No |
| 59 | VCAN |  |  | 4464 | -0.661 | -0.3798 | Yes |
| 60 | NEGR1 |  |  | 4509 | -0.685 | -0.3782 | Yes |
| 61 | TSPAN4 |  |  | 4529 | -0.696 | -0.3717 | Yes |
| 62 | PFN1 |  |  | 4548 | -0.703 | -0.3649 | Yes |
| 63 | RRAS |  |  | 4549 | -0.703 | -0.3546 | Yes |
| 64 | BMP1 |  |  | 4563 | -0.713 | -0.3466 | Yes |
| 65 | THY1 |  |  | 4587 | -0.729 | -0.3404 | Yes |
| 66 | CD274 |  |  | 4766 | -0.837 | -0.3623 | Yes |
| 67 | CALB2 |  |  | 4770 | -0.840 | -0.3506 | Yes |
| 68 | CNN2 |  |  | 4774 | -0.842 | -0.3389 | Yes |
| 69 | ACTG1 |  |  | 4882 | -0.947 | -0.3456 | Yes |
| 70 | SHC1 |  |  | 4885 | -0.948 | -0.3321 | Yes |
| 71 | FLNC |  |  | 4891 | -0.956 | -0.3191 | Yes |
| 72 | ACTN1 |  |  | 4896 | -0.961 | -0.3058 | Yes |
| 73 | CAP1 |  |  | 4920 | -0.984 | -0.2958 | Yes |
| 74 | NF2 |  |  | 4930 | -0.995 | -0.2830 | Yes |
| 75 | EPB41L2 |  |  | 4974 | -1.051 | -0.2759 | Yes |
| 76 | ITGA10 |  |  | 4994 | -1.082 | -0.2637 | Yes |
| 77 | NEXN |  |  | 5098 | -1.288 | -0.2646 | Yes |
| 78 | TGFBI |  |  | 5104 | -1.305 | -0.2465 | Yes |
| 79 | MYH9 |  |  | 5132 | -1.366 | -0.2317 | Yes |
| 80 | VCL |  |  | 5190 | -1.611 | -0.2190 | Yes |
| 81 | ITGA3 |  |  | 5196 | -1.642 | -0.1960 | Yes |
| 82 | LAYN |  |  | 5210 | -1.701 | -0.1736 | Yes |
| 83 | CDH11 |  |  | 5218 | -1.759 | -0.1492 | Yes |
| 84 | EGFR |  |  | 5233 | -1.904 | -0.1240 | Yes |
| 85 | ITGA2 |  |  | 5234 | -1.908 | -0.0961 | Yes |
| 86 | FBN1 |  |  | 5263 | -2.334 | -0.0673 | Yes |
| 87 | ACTG2 |  |  | 5266 | -2.417 | -0.0323 | Yes |
| 88 | MYL9 |  |  | 5272 | -2.509 | 0.0035 | Yes |
Table: GSEA details [plain text format]

  

Fig 2: HALLMARK\_APICAL\_JUNCTION: Random ES distribution      
 Gene set null distribution of ES for **HALLMARK\_APICAL\_JUNCTION**
